# Bladder outlet obstruction; a rare complication of the neglected schistosome, *Schistosoma haematobium*: two case reports and public health challenges

**DOI:** 10.1186/s13104-016-2303-0

**Published:** 2016-11-22

**Authors:** Valirie Ndip Agbor, Tsi Njim, Franklin Ngu Mbolingong

**Affiliations:** 1 Hope Clinic Bamenda, North West Region, Bamenda, Cameroon; 2 Centre for Tropical Medicine and Global Health, University of Oxford, Oxford, Oxfordshire UK; 3 Health and Human Development (2HD) Research Group, Douala, Cameroon; 4 District Hospital Ndu, North West Region, Cameroon

**Keywords:** Schistosomiasis, Neglected tropical diseases, Bladder outlet obstruction, Cameroon

## Abstract

**Background:**

Schistosomiasis is a severe parasitic infestation with debilitating complications and is the third most devastating tropical disease in the world. It is one of the neglected tropical diseases (NTDs) with a high disease-burden. We present two rare cases of bladder outlet obstruction: one which led to a chronic kidney disease and ultimately death and a second which recovered after treatment with praziquantel.

**Case presentations:**

A 72 year old male presented with lower urinary tract symptoms which culminated in an episode of acute urinary retention. The patient had never received preventive chemotherapy with praziquantel. After suprapubic aspiration, the cause of the obstructive uropathy was found to be several mature live worms of *Schistosoma haematobium*. Despite treatment with praziquantel and haemodialysis; we lost the patient due to sepsis from a urinary tract infection. In the second case, a 15 year old male presented with LUTS for a 1 year duration and was diagnosed to have schistosomiasis after eggs of *Schistosoma haematobium* were found in his urine. He was treated with praziquantel.

**Conclusion:**

There are several gaps in the public health policies in place to control this NTD in Cameroon as annual distribution of preventive chemotherapy is inadequate due to inaccessibility of some high-endemic zones and is based on data obtained two decades ago. Population education is insufficient leading to poor health-seeking behaviour. These gaps in public health policies need to be addressed to aid in the overall achievement of the sustainable development goals.

## Background

Schistosomiasis (Bilharziasis) is a parasitic infestation associated with a high degree of morbidity and mortality in third world countries, especially in Africa [1]. Schistosomiasis is endemic in some regions in Africa; of the 261 million people who required preventive treatment for the disease in 2013, 90% resided in this continent. In Cameroon, five million people are estimated to be at risk of infection with the disease, with two million current infections [2]. So far, three species have been identified as causative agents of schistosomiasis in Cameroon: *Schistosoma mansoni*, *Schistosoma haematobium* and *Schistosoma guineensis* (the Lower Guinea species) [3]. Two-third of the cases of schistosomiasis are caused by *S. haematobium*: the species causing urogenital schistosomiasis [4]. In this case series, we present two rare cases of bladder outlet obstruction caused by *S. haematobium*; one which led to a chronic kidney disease and ultimately death and a second which recovered after treatment with Praziquantel.

## Case presentations

### Case 1

A 72 year old African male from Wum, in the far North west region of Cameroon, presented with a 3 year history of lower urinary tract symptoms (LUTS) both obstructive and irritative in nature; and an International Prostate Symptom Score (IPSS) of 22 with a moderate degree of bother, which culminated in an episode of acute urinary retention for which he sought a consult at our health services. The patient like most in his neighbourhood used water from a nearby stream for his baths and claims he had never received any preventive chemotherapy against schistosomiasis.

On examination, the patient was anxious with a tender, renitent hypogastic swelling which increased the zeal to urinate on palpation. A diagnosis of bladder outlet obstruction due to a benign prostate hypertrophy was advocated. Blood samples were collected and a transurethral catheter was placed which relieved the symptoms; with collection of 800 ml of clear urine.

Results of investigations showed: a normal full blood count; prostate specific antigen value of 4.21 ng/dl; normal size and structure of the prostate on ultrasound; serum urea = 321 mg/dl, serum creatinine = 3.2 mg/dl, serum K+ = 6 mEq/l and a microscopic haematuria on urinalysis. A follow up renal ultrasound showed a decrease in the size of the kidneys.

**Fig. 1:**
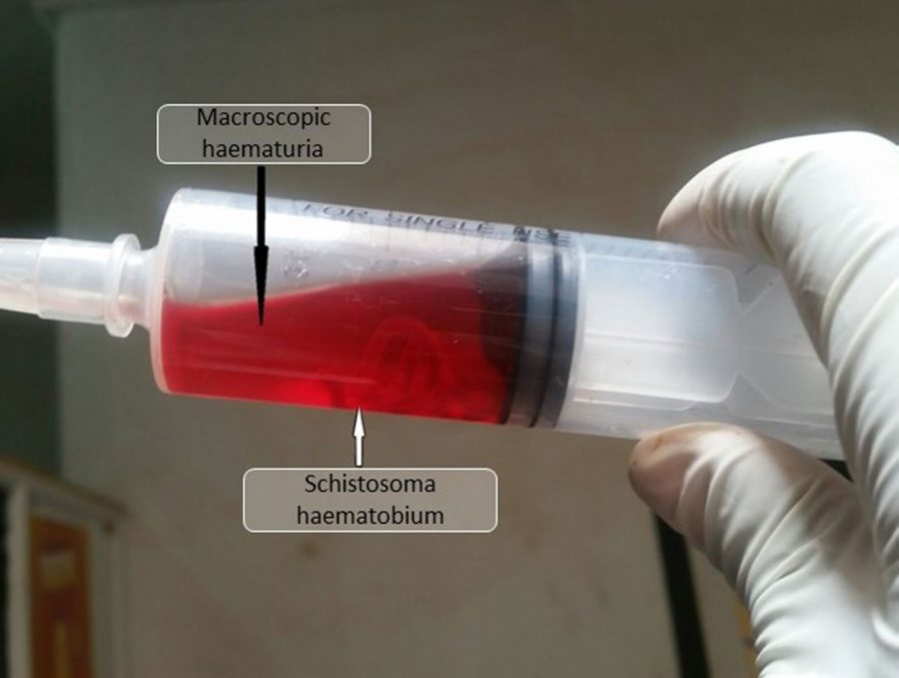
Urine aspirate from supra-pubic puncture; showing adult worms of *Schistosoma haematobium* (*white arrow*) and a macroscopic haematuria (*black arrow*)

On day 2, the patient started having macroscopic hematuria with blockage of urinary catheter. A suprapubic puncture was done with extraction of several live worms which were confirmed to be *S. haematobium* in the laboratory (Fig. 1). The patient was then placed on praziquantel 40 mg/kg and hemodialysis.

Day 6 of hospitalization was marked by the onset of pyuria and fever and the patient was placed on broad spectrum antibiotics and a urine culture requested. We finally lost the patient on day 9 of admission due to sepsis from a urinary tract infection.

### Case 2

A 15 year old African male from Fundong in the North west region of Cameroon, presented with a 1 year history of macroscopic haematuria and dysuria associated with obstructive and irritative LUTS (weak stream, intermittency and frequency), an IPSS of 15 and a moderate degree of bother. He had consulted severally in the health centres in this region by nurses and received multiple treatment with broad spectrum antibiotics. He frequently swam in a river near his home like most children of his age and he had never received any preventive chemotherapy. On examination, the patient had a good general state with normal vital signs. He had mild suprapubic tenderness. Results of investigations revealed a normal FBC; Urinalysis: presence of red blood cells, white blood cells and eggs of S. *haematobium* and serum creatinine: 0.8 mg/dl. He was treated with praziquatel 40 mg/kg which relieved the symptoms. He presented 1 month later for a follow-up visit with no further complaints.

## Discussion

Schistosomiasis, sometimes known as bilharziasis or snail fever is a water borne disease transmitted through skin contact with infested fresh water bodies. According to the World Health Organisation (WHO), schistosomiasis is one of the four neglected tropical diseases (NTDs) controlled through “preventive chemotherapy” intervention (the others include soil transmitted helminthiasis, onchocerciasis and lymphatic filiariasis) [5].

*Schistosoma haematobium* is the second most common isolated species in Cameroon, responsible for up to 95.92% prevalence in some health districts like the Malantouen health district, in the West region [3].

The life cycle of *S. haematobium* begins with the excretion of the egg into fresh water bodies, after which they hatch into the miracidia and penetrate bulinus species (the intermediate host). The parasite emerges from the snails in the larva stage, penetrating the human skin (the definitive host) in contact with the infested water. The larvae then migrate to the lungs and liver. The worms then mature, copulate, and adult female worms deposit their eggs in the pelvic vessels which progressively penetrate urinary and genital system. Eggs deposited during the active phase induce a granulomatous reaction with a resultant fibrosis [6].

Early manifestations of infection include; cough, fever, hepatomegaly, splenomegaly, lymphadenopathies and difficulty in breathing following an initial pruritic papular skin rash (“swimmer’s itch”).

Individuals can develop chronic debilitating complications years after infestation, which include: haematuria, stunting and wasting, bladder cancer, ureteric obstruction, hydronephrosis, urinary tract infections, renal failure and ultimately death [7, 8]. Also, *S. haematobium* is associated with high mortality especially in school-aged children (7–14 years) [9, 10]. Furthermore, 75% of chronically infected females suffer from female genital schistosomiasis which presents with contact bleeding, abnormal discharges, dyspareunia and diminished fertility which is a potential source of stigmatisation and shame [11]. Lastly, the eggs of *S. haematobium* eggs have been reported as Group I carcinogens, causing squamous cell carcinoma; and has also been incriminated as a cofactor for the transmission of the HIV in Africa [12–14].

From the above complications, it is clear that schistosomiasis has a high disease-burden. Despite its public health importance, *S*. *haematobium* has been labeled ‘‘the neglected schistosome’’ [15, 16]. This is true in Africa, and Cameroon in particular, where research on the disease is inadequate. A PubMed search over the last 10 years revealed 13 papers on schistosomiasis from Cameroon, with only two of these papers focused on *S. haematobium*. Furthermore, the failure to control and eradicate schistosomiasis which is one of the NTDs, could be associated with the failure to achieve the Millennium Development Goal (MDG) 6 in most developing countries endemic to the disease in Africa like Cameroon.

Cameroon adopted a plan in 2004 to combat schistosomiasis and soil transmitted helminthiasis (STH) which was implemented in all ten regions by the year 2007. Annual national deworming campaigns were executed with administration of albendazole or mebendazole to school-aged children, whereas the distribution of praziquantel was only to highly endemic for schistosomiasis [17]. In order to control NTDs, the Cameroonian government implemented an integrative approach which involves co-implementing other control intervention measures and co-administering several drugs like ivermectin, praziquantel, mebendazole and albendazole. Since 2009, Cameroon receives assistance from the United States Agency for International Development (USAID) through its NTD Control Program, currently ENVISION program, managed by Response to Intervention (RTI) International. ENVISION facilitates incorporation of national programs and reinforces mass drug administration (MDA) [18].

However, the control data of schistosomiasis in Cameroon is not encouraging. A study carried out in 2013 by Tchuenté et al. showed an increase in transmission foci of schistosomiasis compared to previous data in 1985–1987 [19].

The above program therefore has some gaps in health policies as accentuated by the case reports above. These gaps need to be addressed in order to attain the newly set Sustainable Development Goals (SDGs) amongst which the control and eradication of Schistosomiasis and other NTDs (SDG 3) remains a priority [20].

Due to poor accessibility of most remote areas most individuals inhabiting these zones do not receive treatment. Also, out-of-school children are not reached and preschoolers and adults are not being treated. The annual distribution of praziquantel is therefore defective in controlling and/or eradicating schistosomiasis. Both patients in this case series lived in North West region of Cameroon; in areas with poor accessibility. They and several other members of their community did not receive preventive chemotherapy though they lived in schistosomiasis-endemic zones. Moreso, distribution of prazinquantel is only in highly endemic zones obtained from data 28 years ago. However, there are rapid reinfection rates in high transmission settings due to intense water contact. Control programs are supposed to be coupled with intense monitoring to assess the impact of these interventions. This is however not the case in Cameroon. Hence, newly endemic zones cannot be identified and individuals with the disease go undiagnosed for long periods without access to preventive chemotherapy. Furthermore, there’s the complete absence of environmental control program (snail control and sanitation improvement) which when added to annual drugs distribution may go a long in disease control and eventually disease eradication [21]. Also, the Cameroonian population is deficient in knowledge concerning schistosomiasis including; the mode of transmission, prevention like avoiding wading, swimming, or others contacts with fresh water, and treatment with praziquantel. The patient in case 1 above experienced disabling symptoms for over 3 years and only arrived the hospital at a later stage with an irreversible complication. Population education on the symptoms of the disease and its prevention would lead to better health-seeking behaviors and progress in eventual control of the disease. Finally, the patient in case 2 experienced the disabling conditions of the disease for over 1 year despite several consults at health facilities. This calls for the need for training of healthcare providers on the recognition and management of NTDs like onchocerciasis [22] and schistosomiasis in Cameroon.

## Conclusion

Schistosomiasis is still a major public health problem in Cameroon, associated with significant morbidity and mortality. Individuals, especially school-aged children, adolescents and young adults in endemic zones are at an increased risk to the debilitating conditions of this disease. As shown above, the current health policies have to be revisited and more research has to be done regularly to map out new endemic zones in Cameroon in order to help with the control of the disease and the eventual achievement of the SDGs.

## Abbreviations

NTD: neglected tropical diseases
SDG: sustainable development goals
LUTS: lower urinary tract symptoms
IPSS: international prostate symptom score
WHO: World Health Organisation
MDG: millennium development goals
STH: soil transmitted helminthiasis
USAID: United States Agency for International Development
RTI: response to intervention
MDA: massive drug administration
